# Development and validation of the belonging at work scale: Association with mistreatment and leaves

**DOI:** 10.1371/journal.pone.0346786

**Published:** 2026-04-10

**Authors:** Dayna Lee-Baggley, Hayam Bakour, Bill Howatt, Debra Gilin, Ehsan Etezad

**Affiliations:** 1 Department of Psychology, Saint Mary’s University, Halifax, Nova Scotia, Canada; 2 Howatt HR Consulting, Ottawa, Ontario, Canada; International University - Vietnam National University Ho Chi Minh City, VIET NAM

## Abstract

This research developed the Belonging at Work Scale (BWS), a 7-item, unidimensional measure of work group inclusion focusing specifically on belongingness. Collecting data from 2 Canadian employee samples across 2 studies (*N* = 1535, *N* = 3148), we examined the factor structure, psychometric properties, and group means of the BWS across diverse groups of employees (gender, ethnicity, neurodiversity, sexual orientation). The BWS showed strong reliability as well as configural, metric and scalar invariance across all diverse groups, indicating equivalent fit and applicability. An intersectionality analysis (Study 1) found that women in comparison to men, non-heterosexual individuals in comparison to heterosexual individuals, and participants in intersecting demographic minority groups report less belonging at work on average. Additionally, a greater sense of belonging as measured by the BWS was associated with fewer reports of 10 harmful misbehaviours in the workplace as well as lower rates of taking leaves of absence (Study 2). The development of this scale aims to support organizations in practically measuring their levels of inclusion to ultimately address any identified inclusion-related issues. Study limitations, implications and suggestions for future research are discussed.

## Introduction

In the contemporary landscape of organizational management and human resource development, the concept of inclusion in the workplace has emerged as a central theme of both academic inquiry and practical importance. Inclusion at work is a joint sense of being an integral and valued member of the work group while being appreciated for one’s unique qualities [1]. Increasing diversity within workplaces and the recognition of its inherent benefits has allowed research and application of workplace inclusion to evolve from a mere buzzword into an indispensable imperative in today's workforce [2].

Diversity represents an organization's demographics in terms of groupings such as gender, ethnicity, and sexual orientation [3]. However, the true benefits of diversity (innovation, new ideas, creativity, financial gains) cannot be realized without inclusion [4], that is, diversity is a precursor to true inclusivity at work. It is insufficient to simply incorporate diversity by itself because the outcomes of increasing diversity will depend on how diverse employees are supported at work thus influencing their subsequent ability to thrive in the workplace [5]. In general, inclusion at work can be conceptualized on a continuum representing the degree to which individuals feel a part of important organizational processes, their access to information, connectedness to co-workers, and their ability to participate in and impact the decision-making process [6]. An inclusive workplace respects all perspectives among its employees [7] allowing different groups to support each other to be fully engaged as their complete selves [8]. Inclusion at work is an important concept to understand and measure for several stakeholders. For diverse employees, work provides an avenue to fulfil their professional, social, and financial needs [9]. For the employer, not only have supportive and inclusive work environments been linked with lower levels of anxiety and improved overall mental health [10], but research has also demonstrated direct links with improved levels of performance and an achievement of business goal [10,11]. Additionally, from a legal perspective, anti-discrimination legislation policies mandate the provision of inclusive workplaces and the ability to measure the impact of any such policies implemented [12]. Considering a public health approach, inclusion at work is an important social determinant of health [13] with evidence showing relationships between people’s feelings of organizational inclusion, the need to belong to a larger social group and psychological well-being [14]. Thus, maintaining an inclusive workplace is of crucial importance to organizations.

In addition to the practical importance of inclusion efforts in the workplace, there has been a growing interest in inclusion research in the organizational literature as well [1]. Indeed, inclusion at work and feelings of belongingness have been associated with several important organizational outcomes such as employee health and productivity [15,16]. A meta-analysis by Mor Barak and colleagues [11] found that perceptions of an inclusive workplace climate were consistently associated with only positive work-related outcomes (such as higher job satisfaction and lower turnover intentions). However, there is little agreement in the literature on exactly what constitutes a climate of inclusion in the workplace, with a lack of consistency in the definition and measurement as well as a need for more theoretical grounding. The current research draws upon Optimal Distinctiveness Theory (ODT; [1]) which conceptualizes inclusion to include a degree of belongingness, defined as the need to develop and maintain stable interpersonal relationships. Therefore, with belongingness as the concept of interest, this research aims to extend the inclusion literature by addressing some of the measurement limitations [17] of current instruments (e.g., sample representativeness, scale length, construct and content validity) and developing the brief, theoretically informed, 7-item Belonging at Work Scale (BWS) which has been validated across 2 samples collected from organizations within Canada.

### Inclusion theoretical framework

Utilising optimal distinctiveness theory (ODT) as an organizing framework, Shore and colleagues [1] created a conceptual model of inclusion which they defined as the “degree to which an employee perceives that he or she is an esteemed member of the work group through experiencing treatment that satisfies [their] needs for belongingness and uniqueness”. This definition departs from previous research by focusing on both belongingness and uniqueness as components of inclusion. Belongingness refers to the fundamental need to form and maintain strong interpersonal relationships, while uniqueness emphasizes the value of individual differences [1,18]. Research on inclusion suggests that a sense of belonging is relevant for individuals across visible and invisible forms of diversity as well as across intersectional identities [8]. However, consistent with optimal distinctiveness and social identity perspectives, the salience and expression of uniqueness concerns likely varies both within and between minority and majority demographic groups as shaped by group status, social visibility and identity management demands [1,19–21]. Thus, the current paper focuses on the belongingness component of inclusion.

While both belongingness and uniqueness are critical components for inclusion, belonging has gained more attention over the last several years as organizations have strived to understand how they can make diversity and inclusion efforts more successful. Indeed, at the individual level, the need to be a part of the social whole has long been recognized as core to human psychological well-being [22]. Furthermore, Self-Determination Theory (SDT) proposes three psychological needs which when fulfilled, can intrinsically motivate individuals to perform effectively [23]. In applying SDT and these psychological needs to the workplace context, the need for relatedness, suggests that individuals need to experience meaningful connections with others and feel that they belong within their environment. Outcomes such as job satisfaction, proactivity and organizational commitment, tend to improve when these needs are satisfied emphasizing the importance of belongingness to organizational success [24]. With these considerations as well as applicability of a measure to test the pulse of organizational success as fostering a climate of inclusivity, the current paper centers around measuring inclusion at work with a focus on belonging.

### Feelings of belonging in the workplace

#### Defining belonging.

Belonging is a complex construct [15]. Adler [25] suggests humans are driven to try to fit and belong within the communities they interact. Thus, belongingness is a fundamental human need [18] and has robust impacts on emotional patterns and cognitive processes. Furthermore, a lack of connection to others has been linked to a variety of ill effects on health, adjustment, and well-being [15].

Although belonging occurs as a subjective feeling, the need to belong has been shown at the neural and peripheral biological level [26,27], as well as behaviourally and socially [28]. Biology seems to dictate how sensitive one is to belonging, interacting with social structures and life experiences [29]. Despite its importance, many people struggle to feel a sense of belonging, and those struggles are particularly evident in minorities and other historically equity-seeking groups [30,31]. Belongingness can be categorised as trait (e.g., a core psychological need) and state (e.g., situation-specific senses of belonging). While studies suggest that state belonging is influenced by various daily life events and stressors [32], it appears that multiple processes must converge for a stable, trait-like sense of belonging to emerge and support well-being and other positive outcomes [33,34]. The current study focuses on measuring belongingness as a state specifically relating to the situation at work. Thus, the BWS draws on the various definitions of belongingness and applies them within work scenarios to assess the state of belonging in organizations, and associations with work groups and individual outcomes such as mistreatment and leaves.

#### Measuring inclusion and belonging at work.

Despite the growing emphasis on inclusive workplaces which foster a sense of belonging, substantial gaps remain in both measurement and conceptualization. Indeed, several reviews have highlighted definitional fragmentation and limited theoretical grounding in measure development which include overlapping constructs such as inclusion climate, belonging, psychological safety, diversity climate among others [1,35]. The nuances in construct definition also contribute to the reported compromised psychometric evidence, specifically content and construct validity [17]. Furthermore, existing measures present limitations in scale length, validation across diverse groups as well as frequently relying on student samples that may not reflect real-world organizational complexities [17]. Although employers are advised to measure the impact of their inclusion programmes, a recent study reported that most Canadian organizations only collect statistics on employee use or satisfaction, with minimal data on behaviour changes or impacts on mental health [36]. As a result, many organizations lack understanding on whether their investments are having a meaningful impact on employees. This may not be surprising given that no single measure of belonging exists, rather, studies use an array of different belonging measures across a variety of disciplines.

Thus, in considering the limitations of existing belonging measures, our research aimed to address this gap by developing and establishing the validity and reliability of the brief (7-item), unidimensional, theoretically informed ‘Belonging at Work Scale (BWS).’ We additionally show associations between the BWS and work group climate (mistreatment) as well as employee leaves of absence. The BWS is a measure of work group inclusion focusing on feelings of belongingness developed across two diverse organizational contexts in Canada to ensure a high level of applicability. We developed the BWS by (a) following a deductive item generation process among experts who design and deliver inclusion interventions to workplaces, (b) utilising two independent samples to examine the scale’s psychometric properties and establish criterion validity, (c) examining the performance of the scale across diverse employees to assess its reliability, and (d) assessing the means, psychometric properties, and measurement invariance of the BWS across diverse groups of employees. This study fills a gap in the literature by developing a valid and reliable belonging scale, developed within an organizational context, that can be applied universally across industries and diverse groups to advance the research knowledge in inclusion at work, as well as assessing organizational outcomes of inclusion policies. In addition, our study shows how critical a sense of belonging is to important occupational correlates that indicate an employee’s overall life functioning.

#### Outcomes of belonging.

The need to belong is an essential aspect of fostering diversity and inclusion within an organization [37]. Belongingness plays a crucial role in creating an organizational culture where every employee feels valued, respected, and accepted for who they are [37]. As such, higher levels of belonging at work should be associated with fewer negative behaviour patterns within the work group, as well as improved work well-being of employees.

First, workplaces with a climate that fosters a sense of belongingness should see lower rates of interpersonal mistreatment. Cohesive work groups demonstrate enhanced task performance [38], greater satisfaction with coworkers [11,39], and lower turnover and absenteeism [11] due to a higher sense of security and identity [40]. Indeed, Rubin and colleagues [41] showed that a sense of belonging mediated the relationship between organizational sexism and job satisfaction as well mental health suggesting belongingness as a relevant psychological mechanism for these associations. Similarly, every additional workplace belonging factor professionals endorse predicts lower intentions to leave their profession [42,43], and higher feelings of inclusion correlate with lower burnout, less self-censorship, higher job satisfaction.

On the other hand, a lack of inclusion and belongingness may foster organizational loneliness and isolation which in turn may contribute to misbehaviours in the workplace [44]. Among nurses, workplace ostracism was found to negatively affect attitudes which then predicted negative behavioural outcomes such as counter productive workplace behaviours [45]. Moreover, experimental studies show that social exclusion causes higher levels of stress hormones, higher self-reported stress, and higher negative affect [46]. Additionally, rejection leads to higher levels of retaliation and aggressive intentions [47]. Thus, we anticipate that employees lower on feelings of belongingness will report more interpersonal mistreatment in their work group.

Hypothesis 1: *Employees’ sense of belonging at work will be negatively associated with reports of interpersonal mistreatment in the work group, such as incivility, bullying, and harassment. Employees with a greater sense of belonging will report less mistreatment.*

In addition to the impact on organizational culture, studies have highlighted the numerous positive health impacts of having a sense of belonging such as improved mental health and emotional well-being [15], enhanced persistence [48,49], positive psychosocial outcomes, positive social relationships, occupational success [50], reduced likelihood of absenteeism, decreased feelings of alienation, isolation and disaffection [51], better performance and self-belief in abilities to succeed [52]. Conversely, a lack of belonging (i.e., exclusion) has been linked to higher health risks, lower overall health status [53] and early mortality [54,55]. Leaves of absence from work range from several weeks to years and are predicted by serious mental and physical health struggles [56]. Leaves signify employees reaching diminished professional and personal functioning that indicate poor well-being. A sign of this crisis in functioning is that employees to who take leaves of absence often show detrimental negative effects in their economic, physical, and/or mental health down the road [56]. Further, work leaves involve a protracted period without the positive benefits of work [57] and can involve additional or isolation or stigma due to illness [58]. This may fuel a reciprocal cycle of low feelings of belonging. Thus, we anticipate that this bidirectional relationship creates an association between lower feelings of belonging and a higher frequency of leaves of absence, which has not to our knowledge been reported elsewhere.

Hypothesis 2: *Employees’ sense of belonging at work will be negatively associated with reports of taking a recent leave of absence. Employees who feel greater belonging will be less likely to have taken a leave.*

## Overview of studies

### Study 1 methods

#### Procedure.

Many factors may contribute to feelings of inclusion/belongingness and inclusion itself may be experienced through several diverse lenses (e.g., feeling included with regards to gender, ethnicity, sexual orientation, age, etc.). However, the current BWS was designed to reflect a unidimensional conceptualization of the construct specifically, targeting feelings of belonging [1]. The items were developed based on the assumption that the feeling of inclusion and belonging as an outcome is not multi-faceted regardless of the complex work environment which may contribute to this unitary construct. Additionally, the BWS takes a contextualized and state-based approach to inclusion and belonging that is situation-specific [15].

Two authors of the current study (Lee-Baggley and Howatt) initially generated a list of potential items until they reached a saturation point (45 items) at which no new content seemed unique. The items were derived from the definitions of inclusion and specifically belonging at work [1] as well as the authors’ professional experiences with inclusion-based projects and literature. Following this deductive item generation process, the authors applied their subject matter expertise in examining and rating the items through a structured discussion to facilitate item reduction, resulting in a preliminary scale of 10 items. A second round of content review by PhD students in psychology with training in psychometrics identified that three items included reference to other specific workplace constructs (psychological safety, well-being, and feedback/voice) and were removed to focus the scale just on belonging. This process resulted in the finalized version of the Belonging at Work Scale (BWS) consisting of 7 items rated on a 10-point Likert scale (see Table 1).

**Table 1:** Belonging at Work Scale Items.

On a scale of 1 (low) to 10 (high), How would you rate your level of feeling of being:	
BWS 1	Included in this workplace
BWS 2	Welcomed in this workplace
BWS 3	Safe in this workplace
BWS 4	Like you belong in this workplace
BWS 5	Respected in this workplace
BWS 6	Valued in this workplace
BWS 7	Heard or understood in this workplace

Abbreviations: BWS, Belonging at Work Scale.

The next phase of scale validation included a quantitative survey to examine the scale’s psychometric properties. Participants were recruited through convenience and snowball sampling via the Canadian Standards Association (CSA) members mailing list, social media and targeted contacts. Recruitment efforts targeted human resource, occupational health and safety as well as workplace mental health committee professionals. The survey was completed online through a circulated link from 01/05/2021-01/09/2021. The study was approved by the Research Ethics Board (file # 21–053) and participants provided informed consent prior to completing the survey.

#### Participants.

A total of 2611 participants were recruited, of which 1,535 passed our checks for sufficient completion time, data completeness, and accuracy (missed no more than one of three attention check items, e.g., “Please select ‘strongly agree’ for this item”). The majority age range was 36–40 years old (14.9%). The sample was a majority female (61.8%), Caucasian (76.3%), married (43.1%), neurotypical (75.2%), heterosexual (86.2%), and working full-time (82%) within front-line worker roles (55.7%) but not unionized (64.4%). Most participants reported working for their current employer from 1–2 years (12.5%), 3–4 years (13.2%), 5–6 years (12.3%) and 11–15 years (14.4%), and annual salary of $40,000-$49,000 (16.4%), and working in the private sector—mostly in healthcare (17.2%) or transportation logistics (12.2%) capacities.

#### Measures.

Survey measures included the developed Belonging at Work Scale (BWS) comprised of 7 items rated on a 10-point Likert scale (1 = Low Inclusion “Never Feeling Included”; 10 = High Inclusion “always feeling included”), and demographic information. See Table 1 for items.

## Study 1 Results

### Reliability

The average level of reported feelings of belonging was *M* = 6.68 (*SD* = 2.20), which indicates an average just above the scale midpoint with wide variability. The BWS showed strong internal consistency with a Cronbach’s alpha of  .95 indicating a high degree of inter-item consistency. To examine the performance of the scale across diverse participants, reliability analyses were conducted on the sample for separate categories of gender, ethnicity, neurodiversity and sexual orientation. Alphas (see Table 2) ranged

**Table 2:** Reliability Across Diversity Groups in Study 1 and 2.

Diversity Group		Study 1 *n*	Study 1 α	Study 2 *n*	Study 2 α
**Gender**	Male	546	.947	2356	.957
	Female	949	.947	680	.962
	Non-Binary	7	.946	10	.855
**Ethnicity**	Visible Minority	364	.938	619	.963
	Non-Visible Minority	1171	.950	2478	.955
**Neurodiversity**	Neurodivergent	381	.944	872	.953
	Non-Neurodivergent	1154	.947	2225	.958
**Sexual Orientation**	Heterosexual	1323	.947	2841	.957
	Non-Heterosexual	212	.945	256	.959

*Note.* Overall reliability for study was 1 was α = .95 and for study 2 was α = .96.

from  .94 to  .95 demonstrating high reliability across several diverse groups.

### Factor structure: exploratory factor analysis

An Exploratory Factor Analysis (EFA) was conducted on the 7 developed BWS items using principal components analysis. Bartlett’s test of sphericity [59] indicated that the correlation matrix was not random, χ2(21) = 10,300.16, p < .001, and the KMO statistic [60] was  .92, indicating the items are well-correlated and a common factor seems present. The analysis yielded one factor with an eigenvalue of above one explaining 76.20% of the total model’s variance, and parallel analysis confirmed a one-factor structure because 100 randomly

generated samples with a 95^th^ percentile method had only one component in which the random data had a lower eigenvalue than the actual data [61]. All 7 items showed high loadings on the one factor thus satisfying simple structure criteria [62]. The inter-item correlations ranged between  .53 and  .87 and the internal consistency reliability of this one belonging factor was high, α = .95. As Item BWS 3 (feeling “safe in this workplace”) had a relatively lower inter-item correlation and loading, we scrutinized its construct consistency further. As psychological safety goes hand-in-hand with a sense of belonging and the item may also capture some sense of physical safety as well (capturing some unique variance), we judged it is valuable to retain. Means, standard deviations, factors, percentages of variance explained, factor loadings, communalities and reliability statistics are all presented in Table 3 and inter-item correlations are presented in Table 4.

**Table 3:** Study 1 Exploratory Factor Analysis of Belonging at Work Scale.

			Pattern Coefficient	
**Item**	**M**	**SD**	**Inclusion**	***h*** ^***2***^
**BWS 1**	6.68	2.47	.84	.71
**BWS 2**	7.04	2.37	.90	.82
**BWS 3**	7.33	2.36	.76	.58
**BWS 4**	6.84	2.44	.88	.77
**BWS 5**	6.68	2.58	.92	.84
**BWS 6**	6.18	2.75	.90	.82
**BWS 7**	6.04	2.72	.90	.80
**% of Variance**			76.20%	
**Alpha Coefficient**			.95	
**Eigenvalue**			5.33	

*Note.* Numbers correspond to the item number in the developed BWS. Analysis based on *N* = 1535. *h*^*2*^ *=* item communalities at extraction.

Abbreviations: BWS, Belonging at Work Scale; M, mean; SD, standard deviation.

**Table 4:** Study 1 BWS Inter-Item Correlations.

	BWS 1	BWS 2	BWS 3	BWS 4	BWS 5	BWS 6	BWS 7
**BWS 1**	1.000	–	–	–	–	–	–
**BWS 2**	.81**	1.000	–	–	–	–	–
**BWS 3**	.53**	.61**	1.000	–	–	–	–
**BWS 4**	.75**	.82**	.59**	1.000	–	–	–
**BWS 5**	.70**	.79**	.76**	.76**	1.000	–	–
**BWS 6**	.67**	.74**	.72**	.72**	.85**	1.000	–
**BWS 7**	.67**	.73**	.71**	.71**	.82**	.87**	1.000

Abbreviations: BWS, Belonging at Work Scale.

### Intersectionality analysis

Intersectionality is a concept that describes the overlapping and ‘intersecting’ nature of an individual’s social, cultural, and political identities among many others. To better understand our sample’s unique diversity and experiences, we conducted an exploratory intersectionality analysis to compare individuals based on their self-identified social categorizations, including gender, ethnicity, neurodiversity, and sexual orientation. We examined all 16 possible combinations of binary gender, ethnicity, neurodiversity, and sexual orientation categories (0 = minority, 1 = majority category). We expected that majority males (non-visible-minority, non-neuro-diverse, and heterosexual) would have the highest mean level of belonging. However, we caution that investigating intersectional diversity creates some small intersectional groups (n < 30), and their means cannot necessarily be extrapolated to the broader population.

As shown in Table 5, compared to other possible combinations, participants who identified as male, non-visible minority, non-neurodiverse, and heterosexual (i.e., majority group membership in each category) reported the highest mean level of perceived inclusivity within their work environment. A One-Way Analysis of Variance (ANOVA) was conducted on these intersectionality groups with more than 30 participants to assess group differences on the BWS as an outcome. A significant difference was observed between the groups on their reported feelings of belonging, *F*(15, 1501) = 5.14, *p* < .001, η^2^ = .045. This finding indicates that groups with different combinations of social categorizations and demographics experience different levels of feelings of belonging in the workplace. We used Games-Howell post hoc tests, which correct appropriately for divergent group sizes and are robust to other violations of statistical assumptions [63] to provide further nuance to the results (see Table 5). In addition to majority group males, non-visible minority group males and females who are both non-neurodivergent and heterosexual reported relatively high levels of belongingness.

**Table 5:** Study 1 Intersectionality Group Breakdowns.

Demographics				BWS Score		
Gender	Ethnicity	Neurodiversity	Sexual Orientation	Sample Size	*M*	*SD*
**Male**	**Non-visible Minority**	**Non-Neurodivergent**	**Heterosexual**	**258**	**7.42** ^**a**^	**2.01**
Male	Non-visible Minority	Non-Neurodivergent	Non-Heterosexual	43	6.06	2.41
Male	Non-visible Minority	Neurodivergent	Heterosexual	67	6.79	2.14
Male	Non-visible Minority	Neurodivergent	Non-Heterosexual	22	6.10	2.09
**Male**	**Visible Minority**	**Non-Neurodivergent**	**Heterosexual**	**119**	**7.01** ^**a**^	**1.84**
Male	Visible Minority	Non-Neurodivergent	Non-Heterosexual	17	6.25	2.11
Male	Visible Minority	Neurodivergent	Heterosexual	25	6.79	1.84
Male	Visible Minority	Neurodivergent	Non-Heterosexual	6	7.09	1.94
**Female**	**Non-visible Minority**	**Non-Neurodivergent**	**Heterosexual**	**536**	**6.60** ^**bc**^	**2.23**
Female	Non-visible Minority	Non-Neurodivergent	Non-Heterosexual	38	6.54	2.22
**Female**	**Non-visible Minority**	**Neurodivergent**	**Heterosexual**	**149**	**6.03** ^**b**^	**2.34**
**Female**	**Non-visible Minority**	**Neurodivergent**	**Non-Heterosexual**	**39**	**5.51** ^**b**^	**2.52**
**Female**	**Visible Minority**	**Non-Neurodivergent**	**Heterosexual**	**121**	**7.09** ^**ac**^	**2.03**
Female	Visible Minority	Non-Neurodivergent	Non-Heterosexual	12	5.50	2.32
Female	Visible Minority	Neurodivergent	Heterosexual	35	6.69	2.14
Female	Visible Minority	Neurodivergent	Non-Heterosexual	15	5.65	1.81

*Note.* Games-Howell post hoc tests were applied to determine statistically significant intersectional group means. Bolded lines differed from at least one other group; if means share a superscript letter, they do not differ.

Abbreviations: BWS, Belonging at Work Scale; M, mean; SD, standard deviation.

We supplemented this analysis with a factorial ANOVA with dummy-coded Gender, Female, Visible Minority, Neurodivergence, and Non-Heterosexual factors. This alternative approach found significant main effects for Female [F(1, 1486)=5.40, *p* = .02, η_p_^2^ = .004] and Non-Heterosexual [F(1, 1486)=11.50, *p* < .001 η_p_^2^ = .008] individuals such that female and non-heterosexual participants felt less belonging overall. However, this was nuanced further by a significant higher-order interaction among Gender*Visible Minority*Sexual Orientation [F(1, 1486)=4.73, *p* = .03 η_p_^2^ = .003]. The trend of this interaction shows that heterosexual males feel a high sense of belonging (whether a visible minority or not), White women feel moderately low belonging (whether heterosexual or not), and minority men feel moderately high belonging (whether heterosexual or not), whereas the ‘double jeopardy’ of being both a minority female and non-heterosexual is associated with lowest levels of belonging.

## Discussion

Study 1 developed the Belonging at Work Scale (BWS) through a deductive item generation process and tested the resulting 7-item measure among a large cross-industry sample of employees. The BWS displayed a unidimensional structure, strong internal consistency reliability across diverse participant groups, and variability suggestive of a wide range of subjective belonging. We infer initial construct validity evidence, specifically known-groups validity, from the intersectional group means, which showed that straight white men had the highest sense of belonging. Interestingly, minority heterosexual men and women also showed a high sense of belonging. To our knowledge, our new scale is the first workplace belonging or inclusion scale to be tested on a large, intersectional, and diverse sample of employees and to therefore provide a theoretically-informed tool that is also appropriate and tested for workplace administration.

We acknowledge that the cross-sectional, self-report nature of our data can raise concerns common method bias. In Study 2, to extend our evidence for the BWS, we statistically confirm the unidimensional structure among a second sample, test for invariance among diverse group membership, and gather further convergent validity evidence via associations with work outcomes.

## Study 2 methods

### Procedure

The second study further examined the psychometric properties of the BWS and relationships with hypothesized outcomes within an independent sample. Participants completing their employer’s (a large multi-national automobile organization) contracted workplace assessment for psychological safety were asked for consent to use their data for research purposes. The online survey was distributed through the partner organization’s human resource department. Similar to Study 1, participants in Study 2 completed the Belonging at Work Scale (BWS) and they also reported on their levels of absence (disability, short- and long-term leave) from work and experienced or observed misbehaviors in their workplace. This study was approved by the Research Ethics Board (file # 21–037).

### Participants

A total of 3,781 participants were recruited for the survey, which was available 01/05/2023-01/07/2023. The final sample consisted of 3,148 participants who passed our checks for sufficient completion time, data completeness, and accuracy (missed no more than one of three attention check items, e.g., “Please select ‘strongly agree’ for this item”). The most commonly reported age range of participants was between 46–50 years old (17.7%). A majority of the sample was male (76%), Caucasian (79.6%), heterosexual (91.6%), and non-neurodiverse (71.8%), with English as their first language (90.2%) and working as part of the production team (57.4%). Their average reported salary was $80,000-$89,000 annually (20.1%).

### Measures

Similar to Study 1, participants complete the 7-item BWS. In line with the idea of workplace inclusion climate impacting oneself directly and indirectly [11], participants reported whether or not they had either *experienced or witnessed* a list of 10 items representing misbehaviors in their workplace in the past 12 months (e.g., misunderstandings, conflict, bias, harassment; 0 = Neither Experienced nor Witnessed; 1 = Experienced or Witnessed). Next, participants reported their career incidence of disability leave, short-term leave, and long-term leave (0 = No Leave; 1 = Yes Leave). Lastly, participant demographics were collected.

## Study 2 results

The average level of reported feelings of belongingness on the BWS was *M* = 5.94 (*SD* = 2.35) which was again just above the scale midpoint with wide variability. The rates of participants experiencing or witnessing each misbehavior were as follows: misunderstandings (*n* = 2602, 82.7%), moments of conflict (*n* = 2487, 79.0%), rudeness/incivility (*n* = 2394, 76.0%), ongoing/unresolved conflict (*n* = 2030, 64.5%), covert psychological bullying (*n* = 1659, 52.7%), overt psychological bullying (*n* = 1280, 40.7%), bias or prejudice (*n* = 1482, 47.1%), harassment (*n* = 1271, 40.4%), discrimination (*n* = 1079, 34.3%) and racism (*n* = 879, 27.9%). When examining the different categories of leave of absence, 23.4% reported going on short-term leave (*n* = 738), 2.6% reported going on long-term leave (*n* = 81) and 3.9% reported going on disability-leave (*n* = 122).

### Reliability

The BWS showed strong internal consistency, α = .96. To examine the performance of the scale across diverse participants, reliability analyses were conducted on the sample for separate categories of gender, ethnicity, neurodiversity and sexual orientation. A similar pattern of reliability as Study 1 emerged. Alphas (see Table 2) ranged from  .86 to  .96 demonstrating high reliability across diverse groups.

### Factor structure: confirmatory factor analysis

The hypothesized 1-factor model of the 7-item BWS was tested through a confirmatory factor analysis (CFA). The CFA was conducted using maximum likelihood estimation with FIML for missing values handling in the Lavaan package in R [64]. We allowed or freely estimated the residual correlations among two groups of items that had clusters of similar wording and related conceptual content: BWS items 1, 2 and 4, and BWS items 6 and 7. The following fit indices were considered: Comparative Fit Index (CFI), Root Mean Square Error of Approximation (RMSEA), Tucker-Lewis Index (TLI), Goodness of Fit Index (GFI), and absolute chi-square [59]. Additionally, multigroup CFA was also conducted to test cross-demographic invariance of the BWS for gender (male and female), ethnicity (white and visible minority), sexual orientation (heterosexual and non-heterosexual) and neurodiversity (neurodiverse and non-neurodiverse) categories. Specifically, configural invariance assessed whether the factor structure is similar between groups, metric invariance assessed whether the factor loadings are equivalent across the groups, and scalar invariance assessed whether additionally the intercepts (means) were equivalent [60].

The unidimensional model showed good fit to the data according to most fit indices and CFA results are reported in Table 6 along with factor loadings. The RMSEA value of  .09 is just above most recommendations which suggest ≤ .08 as the cut-off for satisfactory fit [65]. However, many methodologists caution against the use of “a universal cut-off point as the sole means of assessing model fit” [66]. Therefore, for the current unidimensional model, the lower degrees of freedom (*df* = 10) may influence the RMSEA to be borderline, while the remaining model fit indices indicate a good fit.

**Table 6:** Study 2 Confirmatory Factor Analysis of Belonging at Work Scale.

Model	χ2	df	CFI	TLI	SRMR	RMSEA [90% CI]
Unidimensional	263	10	.989	.978	.023	.090 [.080−.099]
BWS Items	M	SD	Skewness	Kurtosis	Factor Loadings	
BWS 1	6.35	2.48	−.40	−.59	.85	
BWS 2	6.75	2.42	−.56	−.35	.85	
BWS 3	5.93	2.72	−.22	−.96	.82	
BWS 4	6.21	2.62	−.35	−.79	.84	
BWS 5	5.88	2.70	−.23	−.95	.92	
BWS 6	5.39	2.75	−.01	−1.10	.90	
BWS 7	5.11	2.72	.12	−1.00	.87	

Abbreviations: BWS, Belonging at Work Scale; CFI, Comparative Fit Index; df, degrees of freedom; CI, confidence interval; GFI, Goodness of Fit Index; M, mean; RMSEA, Root Mean Square Error of Approximation; SD, standard deviation; TLI, Tucker-Lewis Index; χ2, Chi Square.

Additionally, configural, metric, and scalar invariance testing results are reported in Table 7 which show the CFI, TLI, SRMR and RMSEA under increasing constraints of measurement invariance across our key grouping variables (Ethnicity, Gender, Neurodiversity, Sexual Orientation). When sample size is large, chi-square difference tests for invariance analysis are known to inflate small model fit differences [60], we have therefore utilized Chen’s [67] criterion of no degradation of fit (change) across models as large as: CFI = −.01, RMSEA = .001, or SRMR = .015. Table 7 lists the changes from each previously less constrained model in CFI, SRMR, and RMSEA, which by and large showed no or very small (below criterion) fit degradations across more constrained models. Only one comparison exceeds Chen’s [67] criteria: The RMSEA moving from the full-sample (Table 6) to the Ethnicity multi-group configural model (Table 7 first panel) changed from  .090 to  .095, a change of  .005 (which is greater than the  .001 criterion [66]. However, any potential concerns about measurement invariance by ethnicity are alleviated by the fact that further more constrained metric (.087) and scalar (.083) invariance models have improved (lower) RMSEA values compared to the full-sample (.090) and configural models (.095). Results show the fit indices are essentially unchanged from the overall model in Table 6, and that the BWS has an equivalent one-factor structure (configural), loadings (metric), and intercepts (scalar) across the many diverse groups in our sample.

**Table 7:** Multi-Group Confirmatory Factor Analysis: Measurement Invariance Results.

	χ2	df	CFI *(Δ)*	TLI	SRMR *(Δ)*	RMSEA *(Δ)*
*Ethnicity*						
Configural Model	306	20	**.988** *(Δ = −.001)*	.974	**.014** *(Δ=+.001)*	**.095** *(Δ=+.005)*
Metric Model	336	26	**.987** *(Δ = −.001)*	.979	**.023** *(Δ= + .009)*	**.087** *(Δ = −.008)*
Scalar Model	383	32	**.985** *(Δ = −.002)*	.980	**.026** *(Δ= + .003)*	**.083** *(Δ = −.004)*
*Gender*						
Configural Model	273	20	**.989** *(Δ = .000)*	.977	**.014** *(Δ= + .001)*	**.090** *(Δ = .000)*
Metric Model	283	26	**.989** *(Δ = .000)*	.982	**.018** *(Δ= + .004)*	**.080** *(Δ = −.010)*
Scalar Model	330	32	**.987** *(Δ = −.002)*	.983	**.020** *(Δ= + .002)*	**.078** *(Δ = −.002)*
*Neurodiversity*						
Configural Model	274	20	**.989** *(Δ = .000)*	.977	**.014** *(Δ=+.001)*	**.090** *(Δ = .000)*
Metric Model	284	26	**.989** *(Δ = .000)*	.982	**.018** *(Δ=+.004)*	**.079** *(Δ = −.011)*
Scalar Model	287	32	**.989** *(Δ = .000)*	.986	**.018** *(Δ = .000)*	**.071** *(Δ = −.008)*
*Sexual Orientation*						
Configural Model	273	20	**.989** *(Δ = .000)*	.978	**.014** *(Δ=+.001)*	**.090** *(Δ = .000)*
Metric Model	277	26	**.989** *(Δ = .000)*	.983	**.015** *(Δ= + .002)*	**.078** *(Δ = −.012)*
Scalar Model	305	32	**.988** *(Δ = −.001)*	.985	**.016** *(Δ= + .001)*	**.074** *(Δ = −.004)*

Abbreviations: BWS, Belonging at Work Scale; CFI, Comparative Fit Index; df, degrees of freedom; CI, confidence interval; M, mean; RMSEA, Root Mean Square Error of Approximation; SD, standard deviation; TLI, Tucker-Lewis Index; SRMR, Standardized Root Mean Square Residual; χ2, Chi Square.

### Criterion validity: misbehaviors and leave of absence

We used sequential logistic regression analysis to ask whether belonging at work predicts misbehaviors and leaves of absence above demographic control variables: Age, Ethnicity (1 = Non-Visible Minority, 0 = Visible Minority), Gender (1 = Male, 0 = Female), Neurodiversity (1 = Non-Neurodivergent, 0 = Neurodivergent), and Sexual Orientation (1 = Heterosexual, 0 = Non-Heterosexual). Supporting Hypothesis 1, logistic regression analysis results indicated the models adding BWS as a predictor of experienced and witnessed misbehaviors (above and beyond demographic covariates) were all significant and in the expected directions, including misunderstandings (*X*^2^ (1, *N* = 3148) = 343, *p* < .001, R^2^_N_ = .18), moments of conflict (*X*^2^ (1, *N* = 3148) = 382, *p* < .001, R^2^_N_ = .19), rudeness or incivility (*X*^2^ (1, *N* = 3148) = 439, *p* < .001, R^2^_N_ = .21), ongoing or unresolved conflict (*X*^2^ (1, *N* = 3148) = 526, *p* < .001, R^2^_N_ = .22), covert psychological bullying (*X*^2^ (1, *N* = 3148) = 566, *p* < .001, R^2^_N_ = .23), overt psychological bullying (*X*^2^ (1, *N* = 3148) = 485, *p* < .001, R^2^_N_ = .20), bias or prejudice (*X*^2^ (1, *N* = 3148) = 487, *p* < .001, R^2^_N_ = .20), harassment (*X*^2^ (1, *N* = 3148) = 51, *p* < .001, R^2^_N_ = .21), discrimination (*X*^2^ (1, *N* = 3148) = 459, *p* < .001, R^2^_N_ = .20) and bias (*X*^2^ (1, *N* = 3148) = 288, *p* < .001, R^2^_N_ = .13) were all significant. The reported Nagelkerke pseudo R^2^ indicated that the models

containing BWS as a predictor accounted for between 13% and 23% of the total variance (and 11–22% incremental variance after first entering demographic covariates), suggesting that feelings of inclusion contribute moderately to the discrimination between whether employees perceive misbehaviors at work or not. However, the magnitude of this finding does vary by the type of misbehavior reported. Moreover, the BWS was a significant predictor of all misbehaviors at work suggesting that lower levels of belonging predict more reported misbehaviors within the workplace (See Table 8). The odds ratios are all near 0.70, which means that for each one-point increase in belonging an employee perceives, they are around 30% less likely to report seeing or experiencing that misbehavior in their workplace.

**Table 8:** Mistreatment and Leave of Absence as a Function of Belonging, *Study 2.*

Outcome	*B*	*SE*	*df*	*p*	OR
*Misbehaviours*					
**Misunderstanding**	**−.39**	**.03**	**1**	**< .001**	**0.68**
**Moments of Conflict**	**−.380**	**.03**	**1**	**< .001**	**0.68**
**Rudeness or Incivility**	**−.41**	**.02**	**1**	**< .001**	**0.66**
**Ongoing or unresolved conflict**	**−.39**	**.02**	**1**	**< .001**	**0.68**
**Covert psychological bullying**	**−.40**	**.02**	**1**	**< .001**	**0.67**
**Overt psychological bullying**	**−.36**	**.02**	**1**	**< .001**	**0.70**
**Bias or prejudice**	**−.3**	**.02**	**1**	**< .001**	**0.69**
**Harassment**	**−.38**	**.02**	**1**	**< .001**	**0.69**
**Discrimination**	**−.38**	**.02**	**1**	**< .001**	**0.69**
**Racism**	**−.30**	**.02**	**1**	**< .001**	**0.74**
*Leave of Absence*					
**Short-term Leave**	**−.12**	**.02**	**1**	**< .001**	**0.89**
Long-term Leave	−.08	.05	1	.09	0.92
**Disability Leave**	**−.12**	**.04**	**1**	**.001**	**0.89**

*Note.* BWS was the predictor for all presented outcome variables, evaluated with Age, Ethnicity, Gender, Neurodiversity, and Sexual Orientation serving as covariates.

Abbreviations: B, standardized regression coefficient; BWS, Belonging at Work Scale; df, degrees of freedom; SE, standard error; OR, odds ratio.

Additionally, supporting Hypothesis 2, logistic regression analysis results indicated BWS was a significant predictor of leave of absence (controlling for demographic variables), including short-term (*X*^2^ (1, *N* = 3148) = 87, *p* < .001, R^2^_N_ = .04) and disability leave (*X*^2^ (1, *N* = 3148) = 8, *p* = .005, R^2^_N_ = .01) but not long-term leave (*X*^2^ (1, *N* = 3148) = 6, *p* = .44, R^2^_N_ = .01). The reported change in Nagelkerke pseudo R^2^ controlling for demographic covariates indicated that the BWS accounted for an incremental 1% and 3% of the variance in whether participants took short-term, and disability leave, respectively. The BWS was a significant predictor of short-term, and disability leave suggesting that higher levels of inclusion predict less leave (See Table 8).

## Discussion

Study 2 confirmed the BWS as a unidimensional, reliable, and valid scale among a large sample of employees from a global corporation. Further, multi-group analysis demonstrated that the scale is invariant across gender, ethnicity, neurodiversity, and sexual orientation, which supports its application in diverse workplaces. We acknowledge that our brief scale with very high internal consistency also has some correlated item errors, suggesting the items overlap substantially—sacrificing breadth in favor of targeted focus on belonging at work. We also note that while we met criteria [67] for invariance, ethnicity grouping (Caucasian versus not) showed the greatest absolute fit differences as models became more constrained. On closer inspection, this reflected not an issue in functioning of any BWS item/s, but rather *higher mean feelings of belonging* among the minority participants. This was attributable to the company’s very strong focus on diversity, equity, and inclusion, reflected also in their interest in promoting the survey. The BWS scale is functioning well even in such a workplace setting, lending confidence to its use in applied settings. On the other hand, this unique single-organizational context reduces generalizability. This limitation is mitigated by the converging reliability and validity results from Study 1, which was based a large cross-organizational and cross-industry sample. And yet both samples were Canadian. We discuss this potential limitation further in General Discussion.

Finally, a higher sense of belonging was associated with reporting fewer misbehaviors in the workplace and lower incidence of taking short-term leave and disability leave. This is a novel contribution connecting feelings of inclusion with important work and life outcomes. And yet the cross-sectional survey methodology precludes any findings of the causal flow or order between sense of belonging, reporting mistreatment, or taking leaves: Future research will need to disentangle longitudinal patterns. Belonging accounted for a relatively small proportion of incremental variance in leave of absence outcomes suggesting that its effects, while statistically reliable, are modest in magnitude and certainly, belonging is only one of many factors related to both mistreatment and leave-taking.

## General discussion

A sense of belonging is a common core construct that is fundamental to employee well-being and highly relevant regardless of one’s intersectional group membership [8,68]. Reflecting this, the research literature has seen a proliferation of workplace inclusion constructs and measurement scales, many of which are long and multi-faceted, and several of which have limited theoretical basis or validity evidence (9). To our knowledge, none of the existing scales have been tested within diverse workplaces for validity across intersectional group membership, nor have they been created with organizational inclusion benchmarking as a guiding goal of their development. As a result, their universal relevance and utility for this important purpose is unproven.

In this research, we address this critical gap by developing a brief, unidimensional measure of felt belonging at work, the BWS. We found the BWS to be highly reliable, unidimensional and invariant in factor structure, across wide range of diverse groups in two large samples of the working population. In terms of validity, we found that majority group membership was associated with higher mean belonging on the BWS, and that higher felt belonging was associated with a lower reported incidence of ten harmful misbehaviours in the workplace (e.g., harassment, discrimination, racism), and a lower incidence of taking disability and short-term leave in one’s career. The BWS therefore shows promise as a tool to help advance both research on inclusive workplaces and for use in benchmarking the efficacy of organizational inclusivity interventions.

### Theoretical implications

Our results reinforce the fundamental human need that social belonging fulfills [18,23], and demonstrates how the workplace is a critically important community in which to be accepted, connected, and supported through full inclusion and a climate of belonging [11]. Our results indicate that women, non-heterosexual individuals, and people in the minority on more than one demographic dimension (such as visible minority, non-heterosexual men and women) feel less belonging at work on average. The physical and mental stress of exclusion [46,53] may jointly accumulate with illness itself, social engagement, and other contributing personal and work factors [69] to increase the likelihood of work leaves [53]. Leaves, in turn, signify a life functioning disruption that can set off a cascade of future challenges [56], making the fostering of belonging at work an important societal goal.

### Applied implications

A strong sense of belonging marks a critical feature of a healthy and productive workplace [9,10], and our research provides new insight on the collective implications of failing to foster belonging. Inclusivity levels displayed associations with lower rates of harmful workplace factors, that is, experiencing or witnessing instances of misbehaviors (e.g., conflict, bullying, and harassment) in the workplace. We speculate that inclusive workplaces could help reduce incidence of organizational isolation and loneliness [44] and promote less retaliatory coworker and leader responses to aggressive behaviour [47] under stress [46]. This emphasizes the importance of selection, onboarding, and training policies and programs that foster inclusion, to protect employees’ safety and well-being as well as the organization’s sustainability and productivity.

The intersectionality analysis sheds light on the differential feelings of inclusion experienced by individuals based on their social categorizations. A relevant organizational application of the BWS, when it comes to intersectionality, would be for each organization to track feelings of belonging among their employees overall as well as specific considerations for unique subgroup experiences. The BWS, as a very brief measure with equivalent performance across diverse groups of employees, could be administered regularly to benchmark inclusion among the intersectional identities of each unique workforce. Employers can leverage a tool like the BWS to better understand through an intersectional lens how accessible and valuable various employee policies and programs are to different intersectional groupings.

These studies highlight the benefits and importance for employers to take an evidence-based approach when facilitating inclusion and belonging outcomes within their organization. Employers are advised to empirically evaluate the effectiveness of their employee policies and programs (e.g., inclusion initiatives) to validate their impact [37]. However, a significant challenge lies in the absence of valid and reliable tools for measuring the impact of these policies and programs through an inclusion lens. This gap in the availability of suitable measurement instruments underscores the pressing need for brief, reliable, and universally valid measures that can assess the impact of inclusion and a sense of belonging in the workplace. These studies address a significant gap in the literature by providing a valid and reliable belongingness scale that contributes to advancing knowledge in the field of workplace inclusion and enables organizations to evaluate the outcomes of their inclusion policies effectively.

### Limitations and future research

Although the present research contributes a valuable measurement tool, it is not without limitations. First, our survey samples—while including a large catchment of diverse employees—were measured cross-sectionally, such that associations among a sense of belonging and reports of misbehaviors and leaves cannot shed light onto temporal ordering or causal effects. It remains for future longitudinal and intervention research to demonstrate whether improving belonging predicts better employee outcomes.

Second, our collected data occurred with working populations under time constraints, such that we were limited in measuring additional variables that could assess convergent or discriminant validity. Along with these space constraints, in Study 2 we chose to measure specific misbehaviors we hypothesized to relate to a climate of belonging at work (for which, to our knowledge, there is no currently established scale), omitting a more general and validated scale of team rancour (such as the ICAWS, [70]). Further, while Study 1 included a large cross-organizational sample, Study 2 was from a single large diverse industrial workplace, and both were exclusively Canadian samples. Generalizability of our scale’s high reliability, intersectional relevance, and prediction of important work outcomes to other Western and global settings will be interesting and valuable to investigate in future studies considering that even relatively similar national work cultures such as the U.S. and Canada have been found to differ on certain dimensions relevant to well-being [71].

Third, future research should also compare how the BWS performs in comparison to other measurements of inclusion in the workplace. A strength of our study was the intersectionality analysis; however, future research can assess how the BWS performs and compare levels of inclusion on groupings beyond gender, ethnicity, neurodiversity and sexual orientation such as for example different professional and cultural contexts. Moreover, due to the working population of our field sample, the representation of certain diverse groupings was limited thus highlighting an avenue for future research to address. Specifically, in considerations of gender, both our samples were not as diverse beyond the binary of male and female participants which could have limited our ability to capture those inclusion differences across the gender spectrum. Thus, future research could consider gender more intentionally when sampling to better assess inclusion levels and the performance of the BWS.

Lastly, some of the different groupings within the diversity categories we reported on were collapsed for interpretation purposes (e.g., neurodivergent and non-neurodivergent were used instead of the wide range of groupings within the neurodivergent category). Similarly, to the issue of gender, future research can address these categories more intentionally to assess how a wider range of the different groups within each category experience inclusion and belonging.

## Conclusion

Collectively, these findings underscore the pivotal role of inclusivity in fostering a positive, resilient, productive and sustainable workplace environment. In the landscape of today's diverse workforce, the significance of fostering a culture of inclusion, with a prime focus on belongingness, is essential. Belongingness is not only a fundamental human need but also exerts a profound influence on employee and organizational well-being and productivity. Conversely, the absence of belonging can lead to detrimental effects on both individual and organizational levels.

Employers’ efforts to enhance inclusivity may yield not only improved employee well-being but also enhanced organizational outcomes. It is evident that for diversity initiatives to be successful and for organizations to harness the many benefits of a diversified workforce, fostering a culture of belonging is imperative. Future endeavors in this domain should aim at leveraging the BWS to monitor and enhance inclusion strategies and further examining their real-world implications on both individual and organizational outcomes. Through such empirical endeavors, the quest for creating more inclusive, empathetic, and high-performing workplaces can be realized.

To conclude, our study not only introduces a valuable measurement tool in the form of the BWS but also underscores the critical importance of inclusion and belonging in the workplace. The use of scales like the BWS can allow organizations to make thoughtful, data driven decisions about improving employee experience. By recognizing the significance of these factors, organizations can create a more inclusive, diverse, and thriving workplace for their employees, ultimately realizing the benefits of improved individual and organizational outcomes. This research contributes to the ongoing dialogue on inclusion and belonging, offering valuable insights and tools for organizations seeking to enhance their inclusive practices and policies.
